# Competence of non-human primates to transmit *Leishmania infantum* to the invertebrate vector *Lutzomyia longipalpis*

**DOI:** 10.1371/journal.pntd.0007313

**Published:** 2019-04-17

**Authors:** Ayisa Rodrigues de Oliveira, Guilherme Rafael Gomide Pinheiro, Herlandes P. Tinoco, Maria Elvira Loyola, Carlyle Mendes Coelho, Edelberto Santos Dias, Érika Michalsky Monteiro, Fabiana de Oliveira Lara e Silva, Angela Tinoco Pessanha, Andreza Geisiane Maia Souza, Nathália Cristina Lima Pereira, Nelder F. Gontijo, Ricardo T. Fujiwara, Tatiane Alves da Paixão, Renato Lima Santos

**Affiliations:** 1 Departamento de Clínica e Cirurgia Veterinárias, Escola de Veterinária, Universidade Federal de Minas Gerais, Belo Horizonte, Brazil; 2 Fundação de Parques Municipais e Zoobotânica de Belo Horizonte, Belo Horizonte, Minas Gerais, Brazil; 3 Instituto René Rachou, Fiocruz Minas, Belo Horizonte, Minas Gerais, Brazil; 4 Departamento de Parasitologia, Instituto de Ciências Biológicas, Universidade Federal de Minas Gerais, Belo Horizonte, Minas Gerais, Brazil; 5 Departamento de Patologia Geral, Instituto de Ciências Biológicas, Universidade Federal de Minas Gerais, Belo Horizonte, Minas Gerais, Brazil; National Institutes of Health, UNITED STATES

## Abstract

Leishmaniasis is a zoonotic disease of worldwide relevance. Visceral leishmaniasis is endemic in Brazil, where it is caused by *Leishmania infantum* with *Lutzomyia longipalpis* being the most important invertebrate vector. Non-human primates are susceptible to *L*. *infantum* infection. However, little is known about the role of these species as reservoirs. The aim of this study was to evaluate the transmissibility potential of visceral leishmaniasis by non-human primates through xenodiagnosis using the phlebotomine *Lu*. *longipalpis* as well as to identify phlebotomine species prevalent in the area where the primates were kept in captivity, and assess infection by *Leishmania* in captured phlebotomine specimens. Fifty two non-human primates kept in captivity in an endemic area for leishmaniasis were subjected to xenodiagnosis. All primates were serologically tested for detection of anti-*Leishmania* antibodies. Additionally, an anti-*Lu*. *longipalpis* saliva ELISA was performed. Sand flies fed on all animals were tested by qPCR to identify and quantify *L*. *infantum* promastigotes. Eight of the 52 non-human primates were positive by xenodiagnosis, including three *Pan troglodytes*, three *Leontopithecus rosalia*, one *Sapajus apella*, and one *Miopithecus talapoin*, with estimated numbers of promastigotes ranging from 5.67 to 1,181.93 per μg of DNA. Positive animals had higher levels of IgG anti-*Lu*. *longipalpis* saliva when compared to negative animals, prior to xenodiagnosis. Captive non-human primates are capable of infecting *Lu*. *longipalpis* with *L*. *infantum*. Our findings also demonstrate the relevance of non-human primates as sentinels to zoonotic diseases. Several phlebotomine species, including *Lu*. *longipalpis*, have been identified in the area where the primates were maintained, but only one pool of *Lutzomyia lenti* was infected with *L*. *infantum*. This study has implications for public health strategies and conservation medicine.

## Author summary

Visceral leishmaniasis is a zoonotic disease with worldwide distribution. The disease is endemic in several Brazilian regions, including the city of Belo Horizonte, where visceral leishmaniasis is caused by *Leishmania infantum* and transmitted by *Lutzomyia longipalpis*. This study evaluated the competence of non-human primates to infect *Lutzomyia longipalpis* with *Leishmania infantum*. Eight of 52 non-human primates were positive to leishmaniasis by xenodiagnosis, i.e. capable of infecting sand flies, with averages of 5.67 to 1,181.93 promastigotes/μg of DNA. Positive animals had higher levels of IgG anti-*Lu*. *longipalpis* saliva when compared to negative animals, prior to xenodiagnosis. This study highlights the importance of non-human primates in the leishmaniasis cycle, providing information that is relevant for development of better public health strategies, and to conservation medicine.

## Introduction

Visceral leishmaniasis is a zoonotic disease caused by obligate intracellular protozoa of the genus *Leishmania* (order Kinetoplasta: family Trypanosomatidae), which infect macrophages of several mammalian species, including man [1]. There are different species of *Leishmania* that can cause visceral leishmaniasis in the world. *L*. *infantum* (synonym *L*. *chagasi*) has the broadest distribution [2]. The main form of transmission is through the bite of female flies of the subfamily Phlebotominae [1], and the most relevant biological vector in Brazil is *Lutomyia longipalpis* [3].

Although leishmaniasis has a high incidence, morbidity and lethality, it is one of the most neglected zoonotic diseases in the world, affecting mainly deprived human populations from developing countries in tropical areas of the Americas, Asia and Africa, extending to temperate regions of Latin America [1]. Despite its known geographic distribution, leishmaniasis, as others vector born diseases, is a dynamic disease, which transmission circumstances undergo continuous changes dependent on environmental, demographic, and human behavior factors [4].

According to the World Health Organization [1], potential wild reservoirs of visceral leishmaniasis in the New World are wild canids, especially the crab-eating fox (*Cerdocyon thous*), and opossums (*Didelphis marsupialis* and *D*. *albiventris*), although the domestic dog is recognized as the most important reservoir in urban areas [5]. Many other species of wild and synanthropic mammals have also been identified as potential reservoirs, including some species of bats, felines, and neotropical primates [6,7].

To classify an animal as a reservoir, some criteria must be met, among which the capacity of the reservoir host to maintain the availability of parasites in the skin in sufficient numbers to be transmitted to the vectors [1,8]. Xenodiagnosis is an efficient tool to evaluate the capacity of a mammalian host to transmit this pathogen, which characterizes the host as a potential reservoir [9,10]. Although the risk of transmission of leishmaniasis by potential wild reservoirs, such as crab-eating fox [11], wild rabbits [12], maned wolves [9], and bush dogs [9], has already been evaluated by xenodiagnosis, such risk is completely unknown in the case of non-human primates.

Non-human primates can be affected by leishmaniasis, with clinical and pathological manifestations that are similar to those reported in human patients, sometimes remaining asymptomatic [13–15]. However, there are only a few epidemiological studies on the occurrence of leishmaniasis in non-human primates [16], and a complete absence of scientific data on the role they play in transmission of leishmaniasis. This study aimed to evaluate the transmissibility potential of visceral leishmaniasis by non-human primates through xenodiagnoses using the phlebotomine *Lu*. *longipalpis*. In addition, we performed identification of the phlebotomine species prevalent in the area where the primates were kept in captivity and assessed infection by *Leishmania* in captured phlebotomine specimens.

## Methods

### Animals

The experimental protocol employed in this study has been approved by the Ethics Committee on the Use of Animals of the Universidade Federal de Minas Gerais (CEUA/UFMG), under protocol number 94/2013. CEUA/UFMG adheres to the Brazilian legislation (law 11794 –October 8, 2008) under supervision of the Conselho Nacional de Controle de Experimentação Animal—CONCEA.

Fifty two non-human primates kept in captivity at the zoological garden in Belo Horizonte (Brazil) were included in this study, totaling 13 species, 11 neotropical primates and two old world primate species (Table 1). Most of the primates (27/52) were born at the Belo Horizonte zoo, and all of them were housed at the zoo for at least one year prior to this study. A detailed description of the origin of each primate included in this study is provided in a Supplementary Table (S1 Table).

**Table 1:** Species of non-human primates kept at FZB-BH and included at present study.

**Neotropical Primates**			
**Family**	**Scientific Name**	**N**	**Identification**
Atelidae	*Alouatta caraya* *Alouatta guariba* *Lagothrix cana*	4 2 7	01–04 05–06 07–13
Aotidae	*Aotus nigriceps*	2	14–15
Callitrichidae	*Leontopithecus chrysomelas* *Leontopithecus rosalia* *Leontopithecus chrysopygus* *Saguinus imperator*	1 15 2 4	16 17–31 32–33 34–37
Cebidae	*Sapajus paella*	6	38–43
Pitheciidae	*Callicebus nigrifrons* *Pithecia irrorata*	1 3	44 45–47
**Old World Primates**			
**Family**	**Scientific Name**	**N**	**Identification**
Cercopithecidae	*Miopithecus talapoin*	2	48–49
Hominidae	*Pan troglodytes*	3	50–52

### Serological tests

#### Serum sampling

Blood samples of 52 non-human primates were obtained immediately before exposure to the sand flies for xenodiagnosis. The animals were anesthetized with variable protocols according to the species (S2 Table). Blood samples were collected in sterile tubes without anti-coagulant and centrifuged at 2,500 x g for 5 minutes at 4°C. Serum samples were separated and stored at -20°C until serological evaluation.

#### ELISA anti- *Leishmania* (rKDDR and rK39)

ELISA was performed using 96-well-plates (Costar, Cornig, USA) coated with rKDDR (Safetest Diagnósticos, Brazil) or rK39 antigen in carbonate buffer (15 mM sodium carbonate and 34 mM sodium bicarbonate adjusted at pH 9.6) for 24 hours at 4°C. After coated, wells were blocked with 2% PBS-BSA (pH 7.4) for two hours. Serum samples were diluted 1:100 in 0.05% PBS-Tween 20, added to the wells, and incubated for 12 hours at 4°C. Plates were washed five times with 0.05% PBS- Tween 20 solution. Human anti-IgG diluted 1:2500 in 0.05% PBS-Tween 20 solution was added to each well and incubated for 1.5 hours. Plates were then washed, and finally incubated in citrate buffer (0.1 M acid citric and 0.2 M bibasic sodium phosphatase) containing 0.05% *o*-phenylenediamine (OPD) and 0.1% hydrogen peroxide. After ten minutes reactions were stopped with sulfuric acid, and optical densities (O.D.) were measure in an ELISA reader (BioRad 550, Brazil) at 490 nm. Human sera known to be positive or negative were used as positive and negative controls, respectively. Cut off was established at two standard deviations above average O.D. of negative controls.

#### rKDDR Immunochromatographic assay (RAPID test)

The rKDDR Immunochromatographic assay (Safetest Diagnósticos, Brazil) was used according to the manufacturer’s instructions. Briefly, 20 μL of the serum sample was applied without any further processing to the sample well of the test cassette and one drop of PBS buffer (approximately 40 μL) was subsequently added. The result was read visually after 10 to 20 minutes of incubation at room temperature.

#### ELISA anti-*Lu*. *longipalpis* saliva

Salivary glands from *Lu*. *longipalpis* raised at the Hematophagous Insect Physiology Laboratory at the Institute of Biological Sciences at UFMG (LFIH/ICB–UFMG) were dissected and stored in 1% PBS at -80°C. Immediately before use, glands were disrupted by ultrasonication in 1.5 mL conical tubes, and centrifuged at 15,680 x g for 2 minutes. Supernatant was collected and diluted in carbonate buffer (15 mM sodium carbonate and 34 mM sodium bicarbonate, pH 9.6) at concentration of 2.5 salivary glands pairs/mL. ELISA was performed as previously described [17] with few modifications. Human anti-IgG was used as secondary antibody. Positive control was the serum from one chimpanzee (animal 50) of the present study sampled three months after been exposed to sand flies for xenodiagnosis. Wells containing positive serum and no antigen (saliva) were used as negative controls.

### Xenodiagnosis

#### Exposure to sand flies

Xenodiagnosis was performed using 50 four-day-old female *Leishmania*-free *Lu*. *longipalpis* sand flies, raised under controlled conditions at the LFIH/ICB–UFMG. Sand flies were placed in a FleboContainer [9,18], and anesthetized animals were exposed to sand flies directly on the ear for 30 minutes as previously described [9]. Ingurgitation of female flies was assessed visually, and although our parameter of success is at least 70% of females becoming ingurgitated, all animals equal or very close to 100% of female sand flies became ingurgitated. Sand flies were then fed with 50% sucrose for five days at 28°C, frozen, separated in individuals microtubes and stored at -80°C until further analysis.

#### DNA extraction

Ten female sand flies from each animal were randomly selected and individually macerated, homogenized in a 1.5 mL microtube with 50 μL of lysis buffer (0.08 M sodium chloride, 0.16 M sucrose, 0.06 M EDTA, 0.5% SDS, 0.1 M Tris-Cl, pH 8.6) and incubated for 30 minutes at 65°C. Then, 7.1 μL of 8 M potassium acetate were added to the homogenate to a final concentration of 1 M. The material was vortex homogenized and incubated for 30 minutes at 4°C. After incubation, the homogenate was centrifuged at 26,500 x g for 10 minutes. The supernatant was transferred to another 1.5 mL microtube and 100 μL of 95% ethanol were added, then, the mixture was centrifuged at 26,500 x g for 10 minutes. The supernatant was removed and 100 μL of 70% ethanol were added to wash the DNA pellet. The mixture was centrifuged at 26,500 x g for 10 minutes. After complete drying of residual ethanol, the pellet was resuspended in 30 μL of ultrapure water.

#### Identification and quantification of *L*. *infantum* in sand flies

Identification of positive sand flies was performed using qPCR. Primers targeting *Leishmania* sp. minicircle kinetoplast DNA (kDNA) specific for the *donovani* complex were used for qPCR [19]: sense 5’-CTTTTCTGGTCCCGCGGGTAGG-3’, anti-sense 5’-CCACCTGGCCTATTTTACACCA-3’, with final product of 145 base pairs; and primers targeting GAPDH as housekeeping gene: sense 5’-TTCGCAGAAGACAGTGATGG-3’, anti-sense 5’- CCCTTCATCGGTCTGGACTA-3’, with final product of 132 base pairs. qPCR was performed in a final volume of 10 μL, with 0.2 μM of each primer, 5 μL of 1x SYBR Green PCR master mix (Applied Biosystems, USA) and 4 μL of DNA (5 ng/μL). Reaction was performed using an ABI Prism 7500 (Applied Biosystems, USA) and followed initial denaturation at 95°C for 10 minutes, 40 cycles of denaturation at 95°C for 15 seconds and annealing and extension at 60°C for 1 minute.

Ten sand flies from each animal, including both seropositive and seronegative animals, were tested by qPCR in two pools with five sand flies each. Animals that had positive sand fly pools were included in quantitative analysis, and DNA from each individual sand fly that was included in the pools was evaluated separately. Quantification of promastigotes in each sand fly was based on a standard curve established using serial dilutions of *L*. *infantum* pure culture at initial concentration of 10^8^ promastigotes/μL. Calculation of promastigotes/μg of DNA was performed based on Ct of kDNA amplification of each positive sand fly and on slope and y-intersection of *L*. *infantum* standard curve, given the following equation:

$$10^{\left(\text{ct}-\text{y}-\text{intersection})/(\text{slope}\right)}\;\text{x}\;50.$$

### Survey of phlebotomine species in the area where primates were kept in captivity

Phlebotomine capture was performed at 10 sites within the zoo area from February 2014 to February 2015 using traps as previously described [20]. Phlebotomines were identified by morphologic examination based on previously described criteria [21]. DNA samples were extracted from captured phlebotomines and used for nested PCR (LnPCR) amplification of the *Leishmania* SSUrRNA gene [22]. All reactions included positive (20 ng of *L*. *infantum* genomic DNA) and negative controls (target DNA replaced with water). PCR products were sequenced and blasted against the GenBank database for identification of the *Leishmania* species.

### Statistical analysis

Frequencies of positivity of non-human primates by xenodiagnosis were compared using the chi-square test with confidence interval of 95% (p < 0.05). Kruskall-Wallis and Mann-Whitney tests were performed to compare all other data. Agreement between the three serologic tests was calculated by Kappa analysis. All statistical analyses were performed using the Prism software version 7.0 (GraphPad).

## Results

### Serology anti-*Leishmania*

Seven of the 52 non-human primates tested (13.46%) were serologically positive for *Leishmania* spp. using rKDDR as antigen (Table 2). The others two tests (ELISA with rK39 and RAPID test) were a little less sensitive, with 9.61% (5/52) of positivity. However, all serological tests had strong agreement, with kappa coefficient equal to 0.87, using ELISA rKDDR as the main test. One *L*. *rosalia* was positive only at qPCR pool of sand flies, but serologically negative. In total, positive animals included: three *P*. *troglodytes* (100% 3/3), three *L*. *rosalia* (20% 3/15), one *S*. *apella* (16.67% 1/6), and one *M*. *talapoin* (50% 1/2). *P*. *troglodytes* were more predisposed to be serologically positive than the other species included in this study (p = 0.015).

**Table 2:** Serologically positive non-human primates for leishmaniasis with three different serological assays. Legend: ID: individual identification of each animal. NA: not applicable.

Species	ID	rKDDR (O.D.)	rK39 (O.D.)	RAPID test
*Pan troglodytes*	50	+ (0.3232)	– (0.2352)	–
	51	+ (1.2434)	+ (1.5628)	+
	52	+ (1.8469)	+ (2.1753)	+
*Leontopithecus rosalia*	19	+ (0.5595)	+ (0.4301)	+
	27	+ (0.3964)	+ (0.4298)	+
	30*	– (0.0443)	– (0.0605)	–
*Sapajus paella*	43	+ (0.7985)	+ (0.3595)	+
*Myopithecus talapoin*	48	+ (0.3510)	– (0.2157)	–
	**Cut off**	0.2326	0.2563	NA

* Animal positive only by the qPCR of sand flies (xenodiagnosis).

### Xenodiagnosis

**Fig 1:**
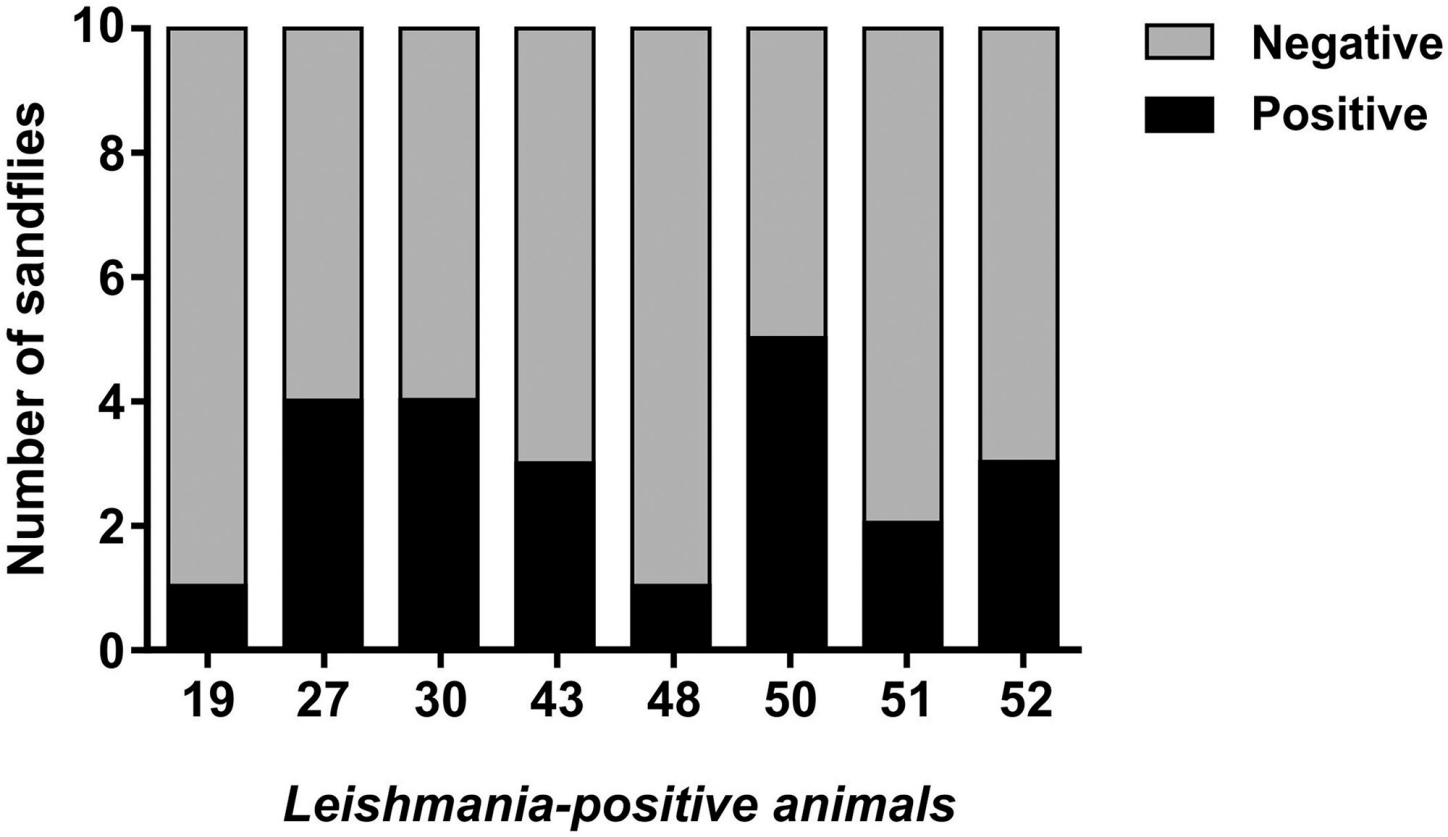
Transmission of *Leishmania infantum* from non-human primates to *Lutzomyia longipalpis*. Ratio of positive and negative sand flies (n = 10) exposed to each of the positive animals, and tested for detection *L*. *infantum* DNA by qPCR.

**Fig 2:**
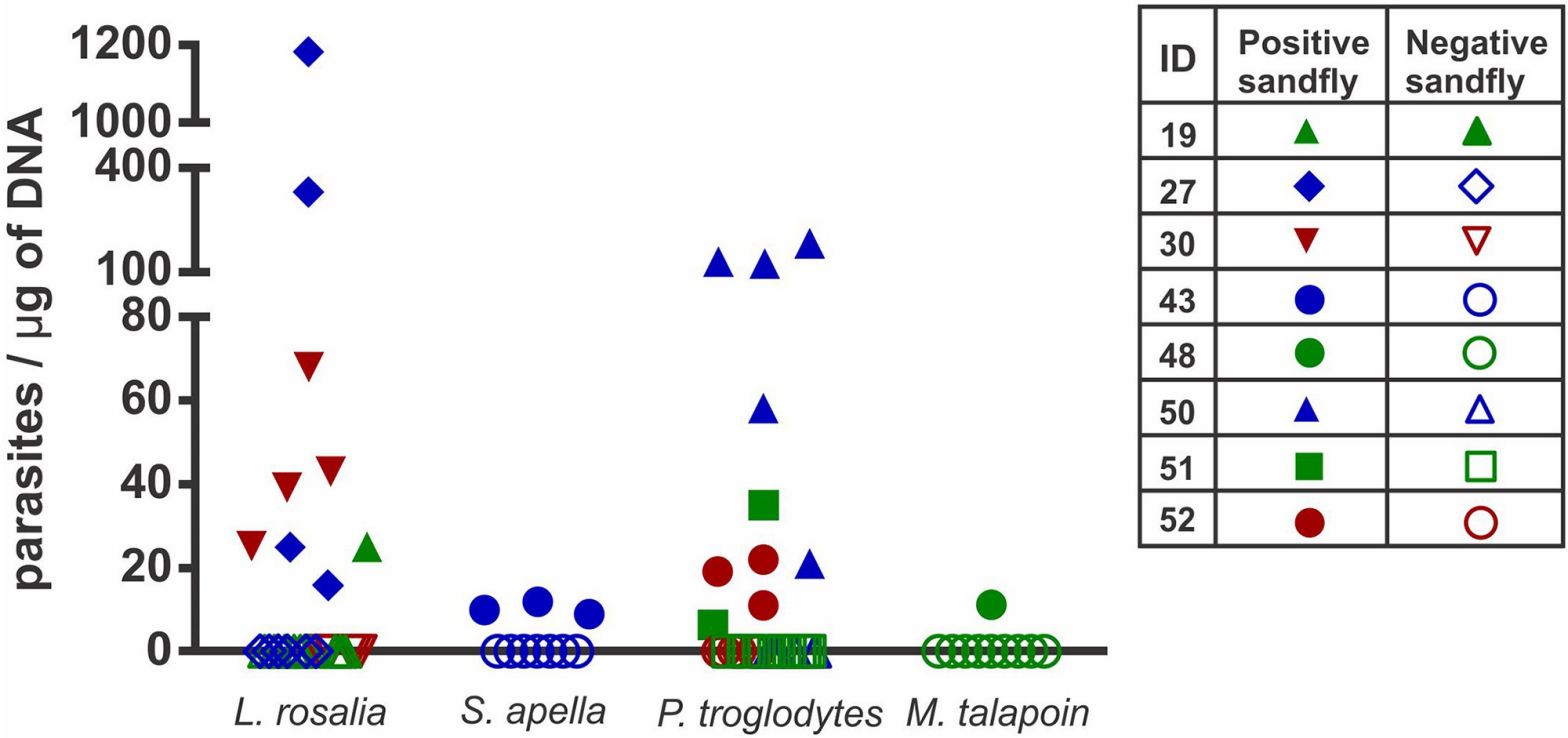
Quantitative analysis of *Leishmania infantum* in *Lutzomyia longipalpis* exposed to non-human primates. Relative quantification of *L*. *infantum* promastigotes in each individual positive sand fly according to the non-human primate species. Symbols refer to individual animals: open symbols indicate negative sand flies, and solid symbols indicate infected sand flies.

Both seropositive and seronegative animals were subjected to xenodiagnosis. All positive animals were capable to infect at least one sand fly, whereas one animal (*Leontopithecus rosalia*) that was serologically negative was also positive by xenodiagnosis (Fig 1). The number of promastigotes/μg of DNA varied from 5.67 to 1,181.93 in positive sand flies (Fig 2).

We observed that *L*. *rosalia* was more efficient than *S*. *apella* to infect sand flies (p = 0.0328), with average of 194 and 10 promastigotes/μg of sand fly DNA, respectively. Although *M*. *talapoin* had one infected sand-fly with also 10 promastigotes/μg of sand fly DNA we could not perform a statistical test because the low number of infected sand fly in this case. *P*. *troglodytes* had an average of 59 promastigotes/μg of sand fly DNA. Fig 2 shows the quantity of promastigotes/μg of DNA of each positive sand fly from each species of the study.

### ELISA anti-*Lutzomyia longipalpis* saliva

**Fig 3:**
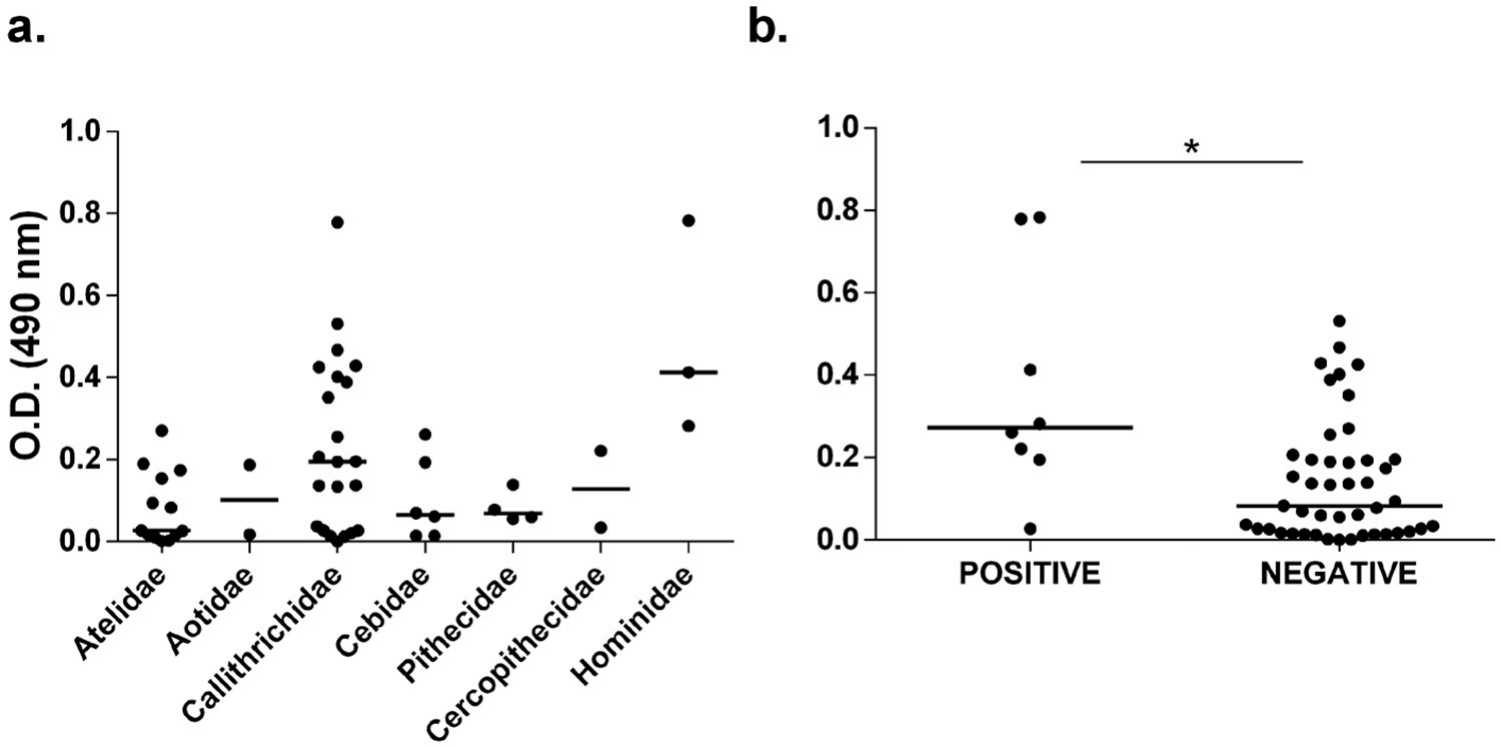
ELISA anti- *Lutzomyia longipalpis* saliva. (A) O.D. from each non-human primate evaluated distributed according their families. (B) O.D. from non-human primates grouped in positive and negative to *Leishmania* spp. by xenodiagnosis. Positive animals had statistically significant higher O.D. values when compared to negative animals (*p = 0.0049 –Mann-Whitney test). Line represents the median and dots indicate individual values.

Although we did not observe significant differences between the O.D. of all families evaluated, animals positive to leishmaniasis had a significantly higher O.D. when compared to negative animals (p = 0.0049) (Fig 3).

### Survey of the phebotomine population in the zoo area

A total of 1,392 phlebotomine specimens were captured, including the following species: *Psathyromyia aragaoi* (Costa Lima, 1912), *Evandromyia bacula* (Martins, Falcão and Silva, 1965), *Evandromyia cortelezzii* (Brèthes,1923), *Lutzomyia ischnacantha* (Martins, Souza and Falcão, 1962), *Lutzomyia lenti* (Mangabeira,1938), *Pintomyia monticola* (Costa Lima, 1932), *Lutzomyia longipalpis* (Lutz and Neiva, 1912), *Pintomyia pessoai* (Coutinho and Barretto, 1940), *Microppygomyia quinquefer* (Dyar, 1929), *Sciopemyia sordellii* (Shannon and Del Ponte, 1927) as detailed in Table 3. Seasonal distribution of captures is detailed in a Supplementary Table (S3 Table).

**Table 3:** Phlebotomines captured within the area of the Zoological Garden in Belo Horizonte (Brazil), from February 2014 to February 2015. Legend: # Pools: indicate the number of pools from each phlebotomine species that were prepared for DNA extraction.

Species	# Specimens			# Pools
	Male	Female	Total	
*Psathyromyia aragoai*	1	0	1	0
*Evandromyia bacula*	0	1	1	1
*Evandromyia cortelezzii*	74	87	161	53
*Lutzomyia ischnacantha*	0	1	1	1
*Lutzomyia lenti*	92	64	156	35
*Lutzomyia longipalpis*	48	23	71	19
*Pintomyia monticola*	27	35	62	23
*Pintomyia pessoai*	485	379	864	82
*Micropygomyia quinquefer*	0	1	1	1
*Sciopemyia sordellii*	0	4	4	4
*Nyssomyia whitmani*	4	8	12	7
Not identified	23	35	58	0
Total	754	638	1392	226

After taxonomic identification of all captured females, 226 species-specific pools with up to 10 phlebotomines each (Table 3) were used for DNA extraction and PCR. These pools included all species with the exception of *Psathyromyia aragaoi*, which had only one male captured. Only one pool of *Lutzomyia lenti* was PCR positive for *Leishmania* spp. The amplified sequence had 97% identity with *L*. *infantum*.

## Discussion

Natural infections and *Leishmania*-associated disease in non-human primates have been occasionally reported, and there is also evidences of infection based on serology and PCR affecting captive and free-living animals [7,13–15,23–26]. Non-human primates have been also extensively used for experimental infections with *Leishmania* spp., especially for vaccinology and clinical or immunopathological studies, with similar outcomes when compared to human patients [27–30]. Xenodiagnosis is the only tool capable of confirming the ability of a potential reservoir to infect the parasite vector. This study describes for the first time the competence of asymptomatic non-human primates to transmit *L*. *infantum* to *Lu*. *longipalpis*, the most important invertebrate vector of visceral leishmaniasis in the New World.

Parasite loads in infected sand flies observed in this study were considered low, although they were similar to parasite loads previously found in phlebotomines fed on asymptomatic or symptomatic *Leishmania*-infected dogs that had averages of 10 and 84 parasites, respectively [31]. However, in that particular study one symptomatic dog infected sand flies with 29,774 parasites. In our study there was also one *L*. *rosalia* that infected a sand fly resulting in a high parasite load (1,181.93 parasites/μg of DNA). Infected sand flies may be classified as “super-spreader” when they carry a high infectious dose (> 600 parasites), which is responsible for epidemic high-disease burden; and “endemic-spreader” with low parasite loads, and responsible for endemic low-disease burden [32]. Therefore, our results suggest that some non-human primate species may play a role in the endemic *Leishmania* cycle, transmitting lower infective doses of parasites to sand flies, favoring the circulation of “endemic-spreader” sand flies, which perpetuate a “mild/asymptomatic mode” of leishmaniasis. This situation is unlikely to favor emergence of clinical disease, but it may result in maintenance of *Leishmania* in a given population within an endemic area [32].

The infective dose of promastigotes in naturally infected sand flies is still unknown, with reports indicating hundreds to thousands promastigotes being required for establishment of infection in a mammalian host [31,33,34]. Some studies indicate that physical obstruction of the sand fly anterior midgut is required for actual transmission of the parasite to mammalian hosts [35,36]. Such obstruction is the result of an association of promastigotes and promastigote secretory gel (PSG) forming a sausage-like plug distending the sand fly anterior digestive tract [36,37]. Obstruction of the anterior gut leads to regurgitation of metacyclic promastigotes during blood feeding, resulting in infection of mammalian host [37]. A minimum number of promastigotes is needed to produce enough PSG to act as a blocking plug. However, the number of promastigotes increase about 16 times within the sand fly [36,37], and sand flies tend to become increasingly parasitized after a second blood meal even if this second meal takes place on an uninfected host [38]. Therefore, it is reasonable to consider that even low parasite loads, as observed in sand flies that fed on non-human primates in this study, could eventually lead to an infective parasite load in an endemic environment. Importantly, we performed qPCR using DNA extracted from sand flies at five days after the blood meal. At five days, ingested blood has been eliminated by the sand fly through defecation [39] so ingested *Leishmania* DNA fragments do not interfere with PCR amplification at that time point. Although later time points may result in higher parasite loads, the PCR technique employed in this study is highly sensitive so sampling of sand flies at 5 days post blood meal was considered appropriate for the goals of this study.

The competence of human individuals to infect sand flies is enhanced in symptomatic and immunocompromised patients [40,41]. Conversely, this tendency is still questionable in domestic dogs [31,42,43]. Previous reports demonstrated that two non-human primates housed at the same institution where this study was done have been diagnosed with symptomatic visceral leishmaniasis: one *Callicebus nigrifrons* in 2008 [13] and one *Gorilla gorilla* in 2016 [15], supporting the notion that captive non-human primates in endemic areas are susceptible to leishmaniasis.

Three serological diagnostic methods were performed in this study: two ELISAs with different antigens (rKDDR and rK39) and one Immunochromatographic (RAPID test) with rKDDR as antigen. Even though ELISA with rKDDR was more sensitive, all three tests had similar results. These results are in agreement with a recent study that demonstrated higher sensitivity and specificity of rKDDR for human or canine sera when compared to traditional serologic protocols [44]. Although the ELISA protocols employed in this study have anti-human secondary antibodies, their results were similar to those obtained with the RAPID test, which have direct interaction of primary antibody from the non-human primate serum with the specific antigen. Importantly, serological tests cannot discriminate infectious animals from those that were previously exposed to *Leishmania*, but are not infectious [9,45]. Therefore, we performed qPCR with a pool of sand flies from each animal, to ensure that positive sand flies could be detected even from serologically negative animals. Interestingly, we observed significantly higher levels of IgG anti-*Lu*. *longipalpis* saliva in positive animals, which corroborate previous studies in dogs and humans, which serology to sand fly saliva has been directly associated with host exposition to sand flies, and increased risk of infection [46–49].

Interestingly, one serologically negative animal (*Leontopithecus rosalia*) was capable of infecting female sand flies. Although in traditional experimental models the peak of parasite load coincides with higher serologic titers [50], non-human primates experimentally infected with *L*. *infantum* often have detectable parasites in the bone marrow or *Leishmania*-induced lesions prior to developing a humoral response [51,52]. Therefore, our hypothesis in this case is that the animal was infected and infectious, but had not yet seroconverted at that time.

Although naturally occurring symptomatic visceral leishmaniasis have been previously reported in non-human primates, it is considered uncommon. There is only one reported case of the disease in a neotropical primate [13], and three recently cases in old world primates, affecting two adults orangutans [14] and one infant gorilla [15]. All of these cases occurred in endemic regions for leishmaniasis. Carneiro and coworkers [53] suggest that neotropical primates have an innate immunological resistance to *L*. *infantum* infection, whereas our results suggest that chimpanzees were predisposed to the infection when compared to other non-human primate species under captivity in an endemic area. All chimpanzees included in this study were positive by xenodiagnosis, and their levels of -*Lu*. *longipalpis* saliva suggested high exposure to sand flies.

Among 1,392 phlebotomine specimens captured within the zoo area, and grouped into 226 pools, only one pool of *Lutzomyia lenti* was PCR positive for *L*. *infantum*. These results may suggest a possible role of *Lu*. *lenti* in the transmission of leishmaniasis. However, these data are insufficient to support this hypothesis since according Killick-Kendrick [54], in order to be considered a biological vector, a given invertebrate species must: (i) feed on humans and animal reservoir species; (ii) support the development of the parasite; (iii) carry parasites that are indistinguishable from the ones isolated from patients; and (iv) be capable of transmitting the parasite through bite.

In conclusion, this study demonstrated for the first time that captive non-human primates might be susceptible to *Leishmania* infection and capable of transmitting the pathogen to sand flies. This study raises awareness regarding the need for improved public health strategies focusing on controlling the vector and the disease. This study also emphasized the importance of non-human primates, wild or captive, as sentinels for zoonotic diseases [55,56], which is highly relevant under a conservation medicine point of view.

## Supporting information

S1 Table Origins and time span of housing at the zoological garden in Belo Horizonte (Brazil) of non-human primates included in the study. (PDF)

S2 Table Anesthetic protocol used during xenodiagnoses for each species of non-human primate. (PDF)

S3 Table Phlebotomine specimens captured within the Zoological Garden in Belo Horizonte (Brazil) from February 2014 to February 2015. (PDF)
