# Supplementary material for: Competence of non-human primates to transmit Leishmania infantum to the invertebrate vector Lutzomyia longipalpis
Source: PLoS Negl Trop Dis. 2019 Apr 17;13(4):e0007313. doi: 10.1371/journal.pntd.0007313 (PMC6488095; doi:10.1371/journal.pntd.0007313)
Supplement: S1 Table — (PDF) [file pntd.0007313.s001.pdf]

S1 Table. Origins and time span of housing at the zoological garden in Belo Horizonte (Brazil) of non-human primates included in the study.

Specie	Popular Name	ID	Gender	Age (years)	Origin	Entrance at FPMZ-BH
Alouatta caraya	Black-and Gold Howler Monkey	01	Female	12	Born at FPMZ-BH/ MG, Brazil	2007
		02	Female	06	Born at FPMZ-BH/ MG, Brazil	2013
		03	Female	06	Born at FPMZ-BH/ MG, Brazil	2013
		04	Male	09	Born at FPMZ-BH/ MG, Brazil	2010
Alouatta guariba	Brown Howler Monkey	05	Male	> 16	Varginha Zoo/ MG, Brazil	2003
		06	Female	> 18	Unknow	2001
Lagothrix cana	Peruvian Woolly Monkey	07	Male	> 03	CETAS from Porto Velho/ RO, Brazil	2016
		08	Female	> 03	CETAS from Porto Velho/ RO, Brazil	2016
		09	Male	> 03	CETAS from Porto Velho/ RO, Brazil	2016
		10	Female	> 03	CETAS from Porto Velho/ RO, Brazil	2016
		11	Female	> 03	CETAS from Porto Velho/ RO, Brazil	2016
		12	Female	> 03	CETAS from Porto Velho/ RO, Brazil	2016
		13	Female	> 03	CETAS from Porto Velho/ RO, Brazil	2016
Aotus nigriceps	Black-headed Night Monkey	14	Female	> 07	CETAS from Porto Velho/ RO, Brazil	2012
		15	Male	03	Born at FPMZ-BH/ MG, Brazil	2016
Leontopithecus chrysomelas	Golden-headed Lion Tamarin	16	Female	> 07	Primatology Center at Rio de Janeiro/ RJ, Brazil	2012
Leontopithecus rosalia	Golden Lion Tamarin	17	Male	06	Born at FPMZ-BH/ MG, Brazil	2013
		18	Male	> 07	Primatology Center at Rio de Janeiro/ RJ, Brazil	2012
		19	Male	05	Born at FPMZ-BH/ MG, Brazil	2014
		20	Male	05	Born at FPMZ-BH/ MG, Brazil	2014

		21	Male	03	Born at FPMZ-BH/ MG, Brazil	2016
		22	Female	04	Born at FPMZ-BH/ MG, Brazil	2015
		23	Male	> 10	Americana Zoo/ SP, Brazil	2009
		24	Male	03	Born at FPMZ-BH/ MG, Brazil	2016
		25	Female	> 07	São Carlos Zoo/ SP, Brazil	2012
		26	Male	03	Born at FPMZ-BH/ MG, Brazil	2016
		27	Male	05	Born at FPMZ-BH/ MG, Brazil	2014
		28	Female	04	Born at FPMZ-BH/ MG, Brazil	2015
		29	Female	05	Born at FPMZ-BH/ MG, Brazil	2014
		30	Female	03	Born at FPMZ-BH/ MG, Brazil	2016
		31	Male	05	Born at FPMZ-BH/ MG, Brazil	2014
Leontopithecus chrysopygus	Black Lion Tamarin	32	Male	> 03	Primatology Center at Rio de Janeiro/ RJ, Brazil	2016
		33	Female	> 03	São Paulo Zoo/ SP, Brazil	2016
Saguinus imperator	Emperor Tamarin	34	Male	09	Born at FPMZ-BH/ MG, Brazil	2010
		35	Male	> 09	CETAS of Rio Branco/ AC, Brazil	2010
		36	Female	08	Born at FPMZ-BH/ MG, Brazil	2011
		37	Male	> 18	CETAS of PortoVelho/ RO, Brazil	2001
Sapajus apella	Margarita Island Capuchin	38	Male	> 05	São Paulo Zoo/ SP, Brazil	2014
		39	Female	12	Born at FPMZ-BH/ MG, Brazil	2007
		40	Female	> 11	CETAS of Belo Horizonte/ MG, Brazil	2008
		41	Male	10	Born at FPMZ-BH/ MG, Brazil	2009
		42	Female	> 13	Primatology Center of Rio de Janeiro/RJ, Brazil	2006

		43	Male	08	Born at FPMZ-BH/ MG, Brazil	2011
Callicebus nigrifrons	Black-fronted Titi Monkey	44	Female	> 02	Wildlife at Divinópolis/ MG, Brazil	2017
Pithecia irrorata	Saki	45	Female	09	Born at FPMZ-BH/ MG, Brazil	2010
		46	Female	12	Born at FPMZ-BH/ MG, Brazil	2007
		47	Female	11	Born at FPMZ-BH/ MG, Brazil	2008
Miopithecus ogouensis	Northern Talapoin Monkey	48	Male	> 29	ZooPark Brasil Nova Iguaçu/ RJ, Brazil	1990
		49	Male	> 27	ZooPark Brasil Nova Iguaçu/ RJ, Brazil	1992
Pan troglodytes	Chimpanzee	50	Male	> 19	Barcelona Zoo, Spain	2000
		51	Male	18	Born at FPMZ-BH/ MG, Brazil	2001
		52	Female	39	Born at FPMZ-BH/ MG, Brazil	1980
