# Supplementary material for: Competence of non-human primates to transmit Leishmania infantum to the invertebrate vector Lutzomyia longipalpis
Source: PLoS Negl Trop Dis. 2019 Apr 17;13(4):e0007313. doi: 10.1371/journal.pntd.0007313 (PMC6488095; doi:10.1371/journal.pntd.0007313)
Supplement: S2 Table — (PDF) [file pntd.0007313.s002.pdf]

S2 Table. Anesthetic protocols employed for each non-human primate species included in the study.

**Neotropical Primates**				
**Family**	**Scientific Name**	**N**	**ID**	**Anesthetic protocol**
Atelidae	*Alouatta caraya*	04	01 – 04	Tiletamine (125mg) + Zolazepam (125mg) 5mg/kg, intramuscular
	Alouatta guariba	02	05 – 06	Midazolam 0.6mg/kg and Ketamine 4-8mg/kg, both intramuscular, and maintenance with isoflurane
	Lagothrix cana	07	07 – 13	Ketamine 5mg/kg, intramuscular, and maintenance with isoflurane
Aotidae	*Aotus nigriceps*	02	14 – 15	Midazolam 0.6mg/kg and Ketamine 4mg/kg, both intramuscular, and maintenance with isoflurane
Callitrichidae	*Leontopithecus chrysomelas*	01	16	Tiletamine (250mg) + Zolazepam (250mg) 4.5mg/kg, intramuscular
	Leontopithecus rosalia	15	17 – 31	17-20: Ketamine 8-10mg/kg, intramuscular, and maintenance with isoflurane.<br>21-31: Tiletamine (125mg) + Zolazepam (125mg) 0.1mg/kg, intramuscular
	Leontopithecus chrysopygus	02	32 – 33	Midazolam 0.5mg/kg and Ketamine 8mg/kg, both intramuscular

	Saguinus imperator	04	34 – 37	34: Midazolam 0.6mg/kg and Ketamine 4-8mg/kg, both intramuscular, and maintenance with isoflurane<br>35-37: Tiletamine (125mg) + Zolazepam (125mg) 0.1mg/kg, intramuscular
Cebidae	*Sapajus apella*	06	38 – 43	Tiletamine (125mg) + Zolazepam (125mg) 6mg/kg and xylazine 0.5mg/kg, both intramuscular
Pitheciidae	*Callicebus nigrifrons*	01	44	Tiletamine (250mg) + Zolazepam (250mg) 4.5mg/kg, intramuscular
	Pithecia irrorata	03	45 – 47	Midazolam 0.5mg/kg and Ketamine 8mg/kg, both intramuscular
**Old World Primates**				
**Family**	**Scientific Name**	**N**	**ID**	**Anesthetic protocol**
Cercopithecidae	*Miopithecus talapoin*	02	48 – 49	Tiletamine (250mg) + Zolazepam (250mg) 4.5mg/kg, intramuscular
Hominidae	*Pan troglodytes*	03	50 – 52	50: Tiletamine (250mg) + Zolazepam (250mg) 13.5mg/kg, intramuscular<br>51-52: Tiletamine (250mg) + Zolazepam (250mg) 13.5mg/kg, intramuscular, and maintenance with propofol, intravenous, and isoflurane

Legend: N: number of the animals from each species. ID: individual identification of each animal.
