# Supplementary material for: Competence of non-human primates to transmit Leishmania infantum to the invertebrate vector Lutzomyia longipalpis
Source: PLoS Negl Trop Dis. 2019 Apr 17;13(4):e0007313. doi: 10.1371/journal.pntd.0007313 (PMC6488095; doi:10.1371/journal.pntd.0007313)
Supplement: S3 Table — (PDF) [file pntd.0007313.s003.pdf]

S3 Table. Phlebotomine specimens captured within the Zoological Garden in Belo Horizonte (Brazil) from February 2014 to February 2015.

Month / Species	February		March		April		May		June		July		August		September		October		November		December		January		February		Total		Total
	♂	♀	♂	♀	♂	♀	♂	♀	♂	♀	♂	♀	♂	♀	♂	♀	♂	♀	♂	♀	♂	♀	♂	♀	♂	♀	♂	♀	♂ e ♀
Psathyromyia aragoai	1	-	-	-	-	-	-	-	-	-	-	-	-	-	-	-	-	-	-	-	-	-	-	-	-	-	1	-	1
Evandromyia bacula	-	-	-	-	-	-	-	-	-	-	-	-	-	-	-	-	-	-	-	-	-	1	-	-	-	-	-	1	1
Evandromyia cortelezzii	3	7	8	6	8	7	7	8	12	6	6	3	3	6	5	7	1	3	13	18	6	11	2	5	-	-	74	87	161
Lutzomyia ischnacantha	-	-	-	-	-	-	-	-	-	-	-	-	-	-	-	-	-	-	-	-	-	-	-	1	-	-	-	1	1
Lutzomyia lenti	10	10	4	1	11	7	9	9	6	6	3	2	6	2	6	5	9	5	5	7	16	8	5	1	2	1	92	64	156
Lutzomyia longipalpis	6	2	2	1	11	7	7	2	9	5	3	3	-	-	5	3	2	-	2	-	1	-	-	-	-	-	48	23	71
Pintomyia monticola	15	16	3	6	3	5	1	5	-	-	-	-	2	-	-	-	-	1	1	1	2	1	-	-	-	-	27	35	62
Pintomyia pessoai	59	34	59	22	152	91	49	55	33	15	7	12	5	7	17	97	4	10	8	3	73	23	9	6	10	4	485	379	864
Micropygomyia quinquefer	-	-	-	1	-	-	-	-	-	-	-	-	-	-	-	-	-	-	-	-	-	-	-	-	-	-	-	1	1
Sciopemyia sordellii	-	2	-	1	-	1	-	-	-	-	-	-	-	-	-	-	-	-	-	-	-	-	-	-	-	-	-	4	4
Nyssomyia whitmani	1	-	-	-	-	-	-	3	-	2	2	2	-	-	1	1	-	-	-	-	-	-	-	-	-	-	4	8	12
* Not identified	4	-	1	-	8	10	1	4	-	-	-	-	2	-	3	9	-	3	2	-	2	6	-	2	-	1	23	34	58
Total	170		115		321		160		94		43		33		159		38		60		150		31		18		1392		1392

* Not identifiable due to the poor quality of the specimens.
